# A Regulator Role for the ATP-Binding Cassette Subfamily C Member 6 Transporter in HepG2 Cells: Effect on the Dynamics of Cell–Cell and Cell–Matrix Interactions

**DOI:** 10.3390/ijms242216391

**Published:** 2023-11-16

**Authors:** Ilenia Matera, Rocchina Miglionico, Vittorio Abruzzese, Giovanna Marchese, Giovanna Maria Ventola, Maria Antonietta Castiglione Morelli, Faustino Bisaccia, Angela Ostuni

**Affiliations:** 1 Department of Sciences, University of Basilicata, 85100 Potenza, Italy; ilenia.matera@unibas.it (I.M.); rocchina.miglionico@virgilio.it (R.M.); v.abruzz@hotmail.it (V.A.); maria.castiglione@unibas.it (M.A.C.M.); 2 Genomix4Life Srl, 84081 Baronissi, Italy; giovanna.marchese@genomix4life.com (G.M.); giovanna.ventola@genomix4life.com (G.M.V.); 3 Genome Research Center for Health—CRGS, 84081 Baronissi, Italy

**Keywords:** HepG2 cells, *ABCC6* silencing, transcriptome analysis, extracellular matrix, adhesion, migration, invasion

## Abstract

There is growing evidence that various ATP-binding cassette (ABC) transporters contribute to the growth and development of tumors, but relatively little is known about how the ABC transporter family behaves in hepatocellular carcinoma (HCC), one of the most common cancers worldwide. Cellular model studies have shown that ABCC6, which belongs to the ABC subfamily C (ABCC), plays a role in the cytoskeleton rearrangement and migration of HepG2 hepatocarcinoma cells, thus highlighting its role in cancer biology. Deep knowledge on the molecular mechanisms underlying the observed results could provide therapeutic insights into the tumors in which *ABCC6* is modulated. In this study, differential expression levels of mRNA transcripts between *ABCC6*-silenced HepG2 and control groups were measured, and subsequently, Gene Ontology (GO) and Kyoto Encyclopedia of Genes and Genomes (KEGG) analyses were performed. Real-Time PCR and Western blot analyses confirmed bioinformatics; functional studies support the molecular mechanisms underlying the observed effects. The results provide valuable information on the dysregulation of fundamental cellular processes, such as the focal adhesion pathway, which allowed us to obtain detailed information on the active role that the down-regulation of ABCC6 could play in the biology of liver tumors, as it is involved not only in cell migration but also in cell adhesion and invasion.

## 1. Introduction

The ATP-binding cassette 6 transporter (ABCC6), one of the ABCC subfamily members, is mainly known because gene mutations are responsible for pseudoxanthoma elasticum (PXE), a rare autosomal recessive disease characterized by a progressive ectopic calcification of elastic fibers in dermal, ocular, and vascular tissues [1,2,3]. ABCC6 is mostly expressed in the liver [4], where it promotes the release of ATP from hepatocytes into the bloodstream [5,6] and contributes to purinergic signaling [7]. Outside the hepatocytes, the released ATP is hydrolyzed by the ENPP1 (ectonucleotide pyrophosphatase/phosphodiesterase 1) to adenosine monophosphate (AMP) and pyrophosphate (PPi), an inhibitor of ectopic mineralization [8]. AMP is then dephosphorylated into adenosine by CD73 (ecto-5′-nucleotidase, Ecto5′NTase), a key player in the regulation of several tumor processes, including invasion, migration, and metastasis [9].

Several studies have investigated the potential associations between ABCC6, cancer development, and multidrug resistance. ABCC6 is involved in the resistance to several anticancer agents, including etoposide, doxorubicin, and daunorubicin [10], and its up-regulation appears to contribute to gemcitabine resistance in human non-small-cell lung cancer and to Nilotinib and Dasatinib resistance in both chronic myeloid leukemia cell lines and primary patient mononuclear cells [11,12]. Moreover, in head and neck squamous cell carcinoma cancer stem cells, its down-regulation following valproic acid treatment seems to contribute to reduced chemoresistance [13].

Trujillo-Paolillo et al. investigated the expression of genes related to pharmacogenetics in osteosarcoma and discovered that tumors from metastatic patients had a higher expression of *ABCC6* than tumors from non-metastatic patients [14]. The overexpression of *ABCC6* is frequently associated with a bad outcome in patients with stomach cancer [15] and highly aggressive tumors such as high-grade serous ovarian cancer [16]. In contrast, *ABCC6* expression was associated with survival of lung adenocarcinoma in [17].

Contrary to previous findings, it was also revealed that *ABCC6* down-regulation is associated with enhanced treatment resistance in some cancers. For example, cisplatin-resistant ovarian cancer cell lines and colorectal cancer non-responders to treatment have lower ABCC6 expression than responders [18,19]. *ABCC6* down-regulation has also been associated with the development and progression of pancreatic ductal adenocarcinoma [20].

Regarding the role of ABCC6 in hepatocellular carcinoma, a recent study demonstrated that low expressions of *ABCC6* and other ABCC transporters are correlated with poor prognoses in liver hepatocellular carcinoma, identifying these proteins as potential diagnostic markers [21]. Zhao et al. reported that *ABCC6* is down-regulated in HCC tumor tissues and correlates with favorable outcomes in patients with HCC [22].

In hepatocarcinoma HepG2 cells, *ABCC6* knockdown and its pharmacological inhibition by Probenecid result in cytoskeleton reorganization and the down-regulation of CD73 expression. The restoration of the normal morphology of filopodia and migration rate in HepG2 upon addition of ATP or adenosine suggests that this effect is most likely caused by a decrease in the extracellular ATP and adenosine pool through a modification of ABCC6-mediated ATP efflux [7,23,24] under the control of the PI3K/AKT signaling pathway, which is down-regulated in *ABCC6* knockdown cells [25,26]. Furthermore, through providing ATP to the extracellular purine pool, ABCC6 appears to play a significant role in regulating migration into Caco2 colon cancer cells [27].

Cell migration, invasion, and adhesion are crucial steps in cancer progression. These processes involve a number of cellular mechanisms led by cytoskeleton dynamics, as well as molecular changes such as the production of adhesion and proteolytic enzymes [28,29]. An altered expression of cell adhesion molecules, including cadherins and integrins, can enhance tumor cell adhesion to the extracellular matrix (ECM), thus promoting invasion and metastasis [30,31].

The purpose of this study was to understand the role of the ABCC6 transporter in the biology of hepatocarcinoma. By using high-throughput RNA-Seq technology, the impact of *ABCC6* silencing in HepG2 cells was explored. The dysregulation of the focal adhesion pathway, shown by KEGG functional enrichment analysis, demonstrated that ABCC6 transporter activity is implicated in cell–ECM adhesion. The silencing of *ABCC6* may lessen the aggressive phenotype of HepG2 cells by preventing EMT and decreasing cell motility and invasion.

## 2. Results

### 2.1. Whole Transcriptome of the ABCC6-Silenced HepG2 Cell Line

Whole transcriptome RNA sequencing was performed by next-generation sequencing in *ABCC6*–shRNA (SH–*ABCC6*) and Scramble–shRNA (SCR–RNA) cells to highlight possible gene differences between the two conditions. After filtering out low-quality reads and trimming the adaptors, the obtained reads were aligned against the human genome reference (HG38—Release 37 (GRCh38.p13)) [32].

More than 16,638 expressed normalized genes were identified and quantified between samples. Principal components analysis (PCA) suggested good mRNA expression patterns in the two group of cells.

**Figure 1:**
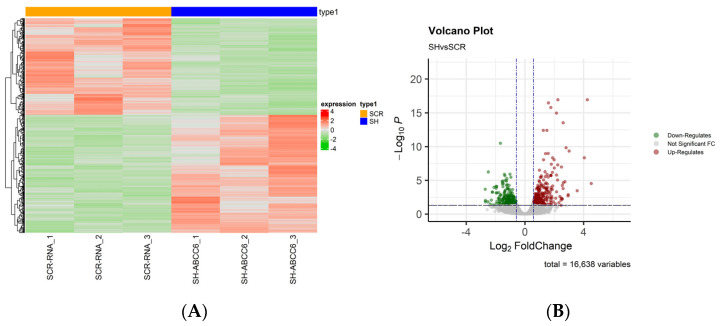
The analysis of differentially expressed genes. (**A**) A heatmap of differentially expressed genes. Red indicates that the expression level of the gene is relatively up–regulated, and green indicates that the expression level of the gene is relatively down-regulated. (**B**) Volcano plot of differentially expressed genes, where in black is shown the pvalue cutoff, instead in blue, log2(Fold-change) cutoff.

To investigate the overall mRNA expression differences between the two groups, hierarchical clustering analyses were performed. Samples showed expression heterogeneity among two groups, suggesting a molecular diversity that is reflected by mRNA expression (Figure 1A). A Volcano plot was constructed to show the differentially expressed genes between the two groups (Figure 1B).

In detail, our differential expression analysis revealed 476 statistically significant (padj ≤ 0.05) genes between the two groups (SHvsSCR). Among them, 256 genes were significantly (padj ≤ 0.05 and |FC| ≥ 1.5) up-regulated, and 208 genes (padj ≤ 0.05 and |FC| ≤ −1.5) were significantly down-regulated. The raw data and the normalized count of genes identifies are available at the ArrayExpress repository under accession number: E-MTAB-13154.

The top 10 differentially expressed genes that were found to be up-regulated and down-regulated in *ABCC6*-silenced HepG2 cells are shown in Table 1.

### 2.2. Functional Enrichment Analysis of Differentially Expressed Genes

To identify the active subnetworks that were enriched by the obtained differentially expressed genes, we used the pathfinder tool. We chose to use two functional enrichment analysis by selecting the Gene Ontology (GO) and Kyoto Encyclopedia of Genes and Genomes (KEGG) databases, respectively.

**Figure 2:**
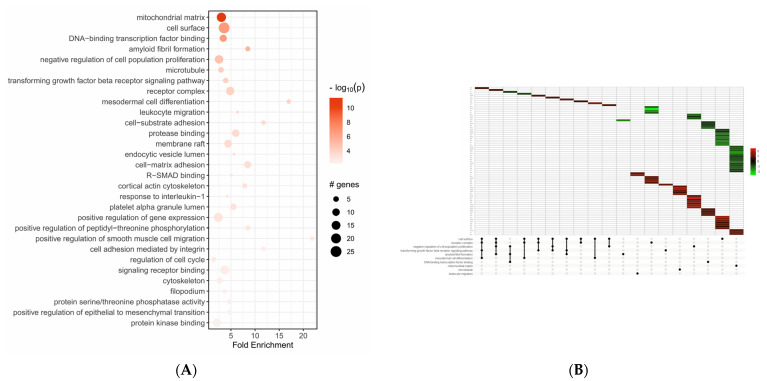
GO enrichment analysis of differentially expressed genes. (**A**) Enriched plot of GO analysis of up- and down-regulated genes. The top 30 terms are shown. (**B**) GO UpSet plot combines intersections of enriched terms below *x*-axis and bar plot of the number of genes in the corresponding intersections showing relative log2FoldChange. In the various intensities of green and red we see genes down-regulated and up-regulated.

GO enrichment analysis was performed to analyze the functions of the differentially expressed genes; the top 30 GO terms are displayed in Figure 2. The enriched GO term of up- and down-regulated differentially expressed genes were mainly mitochondrial matrix (GO:0005759), cell surface (GO:0009986), and DNA–binding transcription factor binding (GO:0140297), as shown in Figure 2A. Moreover, as shown in Figure 2B, in the top enriched GO terms, we found the most up- and down-regulated differentially expressed genes.

**Figure 3:**
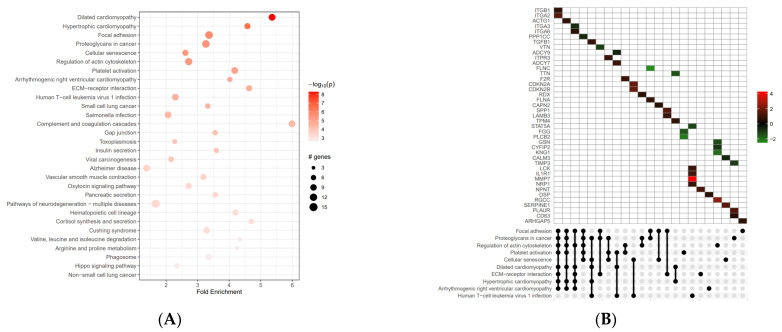
Our KEGG pathway enrichment analysis of differentially expressed genes. The results of our analysis of up-regulated and down-regulated genes are shown, along with the top 30 pathways. (**A**) The up- and down-regulated differential genes are mainly concentrated in the protein processing of dilated cardiomyopathy and focal adhesion. (**B**) KEGG UpSet plot combines intersections of enriched terms below *x*-axis and bar plot of the number of genes in the corresponding intersections showing relative log2FoldChange. In the various in-tensities of green and red we see genes down-regulated and up-regulated.

KEGG pathway enrichment analysis showed that the differentially expressed genes participated in 91 statistically significant pathways. The enriched KEGG pathways were mainly dilated cardiomyopathy (hsa05414), focal adhesion (hsa04510), and proteoglycans in cancer (hsa05205), with 10 genes (up- and down-regulated) mainly involved, as shown in Figure 3 and Table 2 (data extracted from Supplementary Table S1).

### 2.3. The Silencing of ABCC6 Changes the Expression of Some Proteins Involved in Cell–Cell and Cell–Matrix Interactions

The dysregulation of proteins responsible for cell–cell and cell–ECM interactions led us to investigate the possible involvement of ABCC6 in these dynamics.

**Figure 4:**
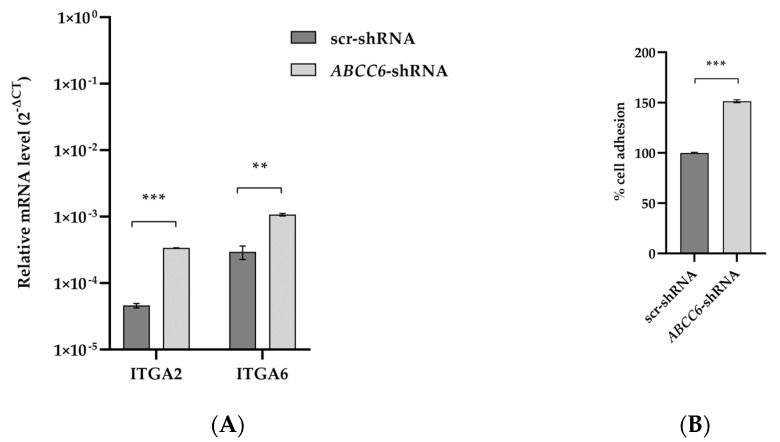
Expression levels of ITGA2 and ITGA6 transcripts and adhesion of *ABCC6*-silenced HepG2 cells to Matrigel. (**A**) RT–PCR results are expressed as the 2^−ΔCt^ and are presented as the mean ± SEM of at least three independent experiments. β-actin was used as a reference gene. Statistical analysis was performed using Student’s *t* test; ** *p* < 0.01, *** *p* < 0.001. (**B**) Adhesion of scr–shRNA and ABCC6–shRNA cells to Matrigel. The percentage of adherent cells was plotted as a percentage of total cells. Data are mean of three independent replicates with three biological replicates (independent clones) per experiment. Results were analyzed using Student’s *t* test; *** *p* < 0.001.

Integrin-mediated adhesion and signaling dysregulation are precursors in cancer etiology. Integrins contribute to ECM remodeling and facilitate cancer cell colonization in new metastatic sites. Among the top pathways affected by *ABCC6* gene silencing, the focal adhesion pathway shows increased expression levels of the driver genes ITGA2 and ITGA6. The adhesion of *ABCC6*-silenced cells to Matrigel increased by 50% (Figure 4).

**Figure 5:**
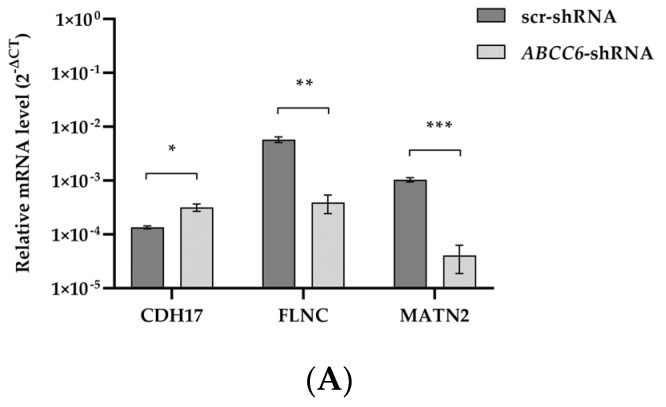

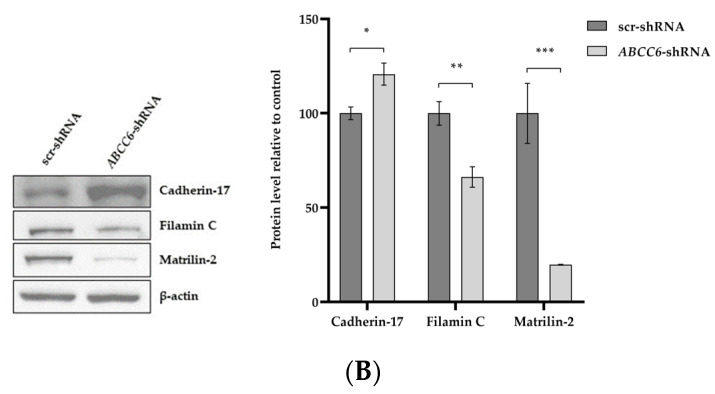
The expression levels of CDH17, FLNC, and MATN2 in *ABCC6*-silenced HepG2 cells. (**A**) Results are expressed as the 2^−ΔCt^ and are presented as the mean ± SEM of at least three independent experiments. β–actin was used as a reference gene. Scrambled cells (scr–shRNA) were used as controls for *ABCC6* knockdown (*ABCC6–shRNA* cells). (**B**) Representative Western blot and densitometric analysis of the immunoreactive bands. The protein levels were normalized with β-actin content and refer to that of scr–shRNA cells set to 100%. Data are presented as mean ± SEM of at least three independent experiments. Statistical analysis was performed using Student’s *t* test; * *p* < 0.05, ** *p* < 0.01, *** *p* < 0.001.

RT-qPCR and Western blotting were performed to evaluate the effect of *ABCC6* silencing on some liver cancer-related genes. According to our transcriptomic analysis, the expression levels of FLNC and MATN2 are significantly lower; on the contrary, the expression level of CDH17 is weakly increased in silenced cells (Figure 5).

### 2.4. ABCC6-Silenced Cells Modify Their Clonogenic Potential and Ability to Invade the Extracellular Matrix

**Figure 6:**
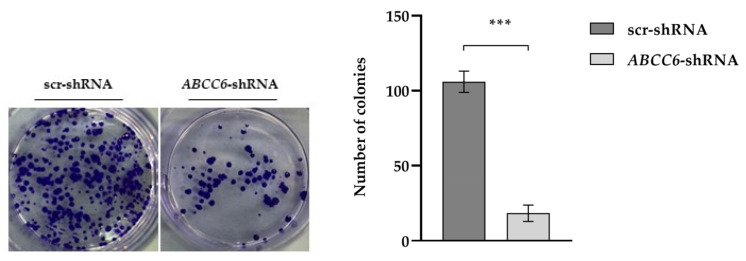
Clone formation assay. Images show the colonies formed by control HepG2 (scr–shRNA) and *ABCC6*-silenced HepG2 cells. Data are presented as the mean ± SEM of three independent experiments. Statistical analysis was performed using Student’s *t* test; *** *p* < 0.001.

A colony formation assay demonstrated that the colony numbers of the *ABCC6–shRNA* HepG2 cells were significantly decreased compared with the control group, thus showing that they are less aggressive than scrambled ones (Figure 6).

**Figure 7:**
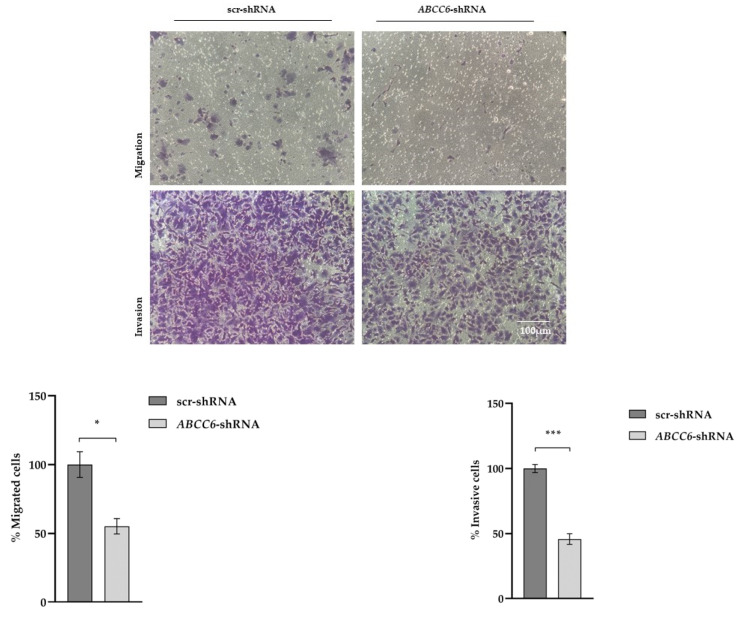
Migration and Matrigel invasion assay of scr–shRNA and ABCC6–shRNA HepG2 cells using a transwell system. Purple represents migrating and invaded cells dyed with crystal violet (magnification ×20, FLoidTM Cell Imaging Station). Data are presented as the mean ± SEM of three independent experiments. Statistical analysis was performed using Student’s *t* test; * *p* < 0.05; *** *p* < 0.001.

In order to verify whether the silencing of the *ABCC6* gene can also compromise the ability to invade the ECM, the transwell assay with Matrigel was performed. The silenced cells were considerably less invasive than the control cells (Figure 7). Without Matrigel, according to the results of a previous wound healing assay [7], *ABCC6–shRNA* cells migrated more slowly.

It is known that matrix metalloproteinases are responsible for the destruction of the ECM and the growth of cancer [33]. Both cell lysates and media were analyzed for the presence of gelatinolytic active metalloproteinases. The expression levels of MMP9 and MMP2 were significantly reduced in *ABCC6*-silenced HepG2 cells. The gelatinases are secreted enzymes. Media from scr–shRNA cells and *ABCC6–shRNA* cells were subjected to gelatin zymography. The SDS–gelatin gel zymogram demonstrated three gelatinolytic bands, including gelatinase B (92 kDa, gelatinase/MMP9) and gelatinase A (72 kDa, gelatinase/MMP2). The gelatinolytic intensity was stronger in the control cells than in the silenced cells.

**Figure 8:**
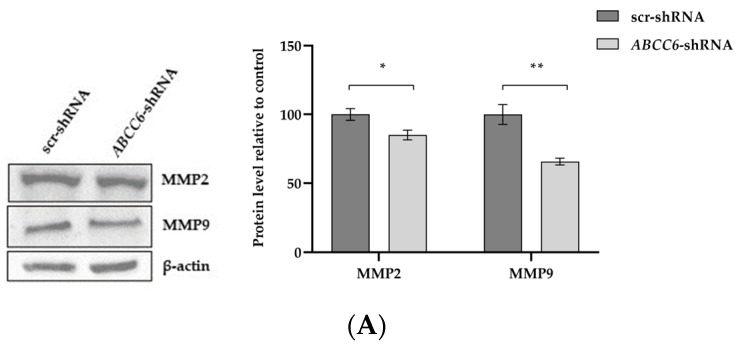

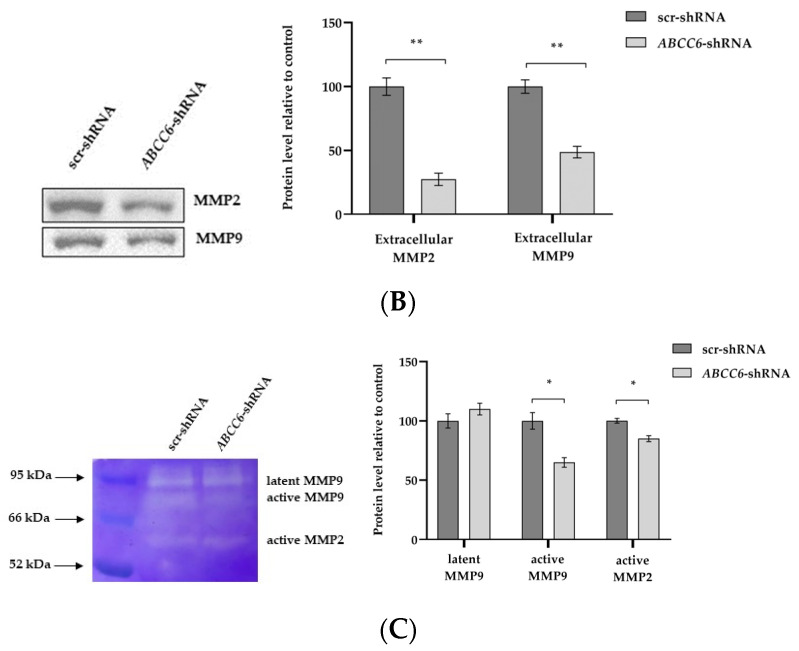
Expression levels of MMP2 and MMP9 in ABCC6-silenced HepG2 cells. (**A**) Representative Western blot of scr–shRNA cells and ABCC6–shRNA cell lysates. Scr–shRNA cells were used as controls for ABCC6 knockdown. Densitometric analysis of the immunoreactive bands performed in three independent experiments. The protein levels were normalized with β-actin content. (**B**) Expression level of extracellular MMP2 and MMP9 in cell media. The protein levels in the culture media of scrambled and silenced cells were normalized with total protein content. Data shown refer to those obtained on the culture medium of the scrambled cells set to 100%. Data are presented as the mean ± SEM of three independent experiments. (**C**) Gelatin zymography gel comparing MMP2 and MMP9 production in culture media of scr–shRNA cells and ABCC6–shRNA cells. Densitometric analysis of the bands were performed in three independent experiments. The protein levels were normalized with total protein content. Data were normalized to scr–shRNA cells set to 100%, and are presented as the mean ± SEM of three independent experiments. Statistical analysis was performed using Student’s *t* test; * *p* < 0.05; ** *p* < 0.01.

As demonstrated by gelatin zymography, the activated enzymes were released from the cells into the medium. Control scrambled–transfected cells showed a reduced activity of both metalloproteinases (Figure 8).

### 2.5. ABCC6 Silencing Dysregulates Genes Involved in the Epithelial–Mesenchymal Transition

**Figure 9:**
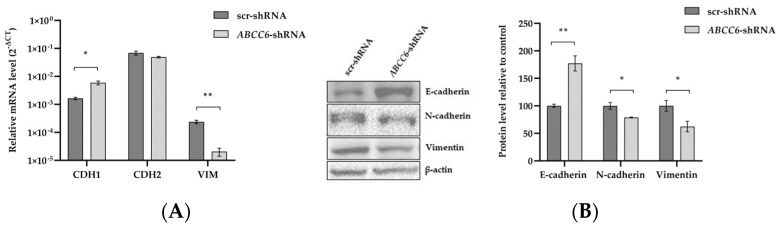

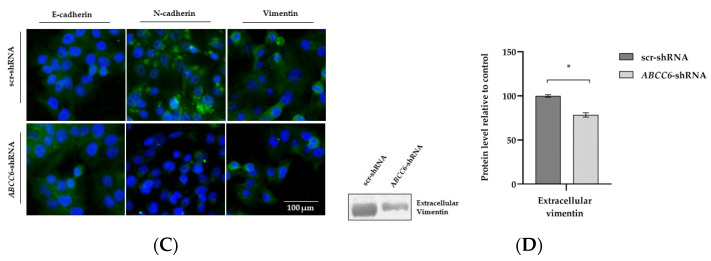
Expression analysis of EMT proteins. (**A**) Relative mRNA expression levels of Vimentin, N–cadherin, and E–cadherin genes in scr–shRNA and *ABCC6–shRNA* cells. Results are expressed as the 2^−ΔCt^ and are presented as the mean ± SEM of at least three independent experiments. β-actin was used as a reference gene. (**B**) Representative Western blot of scr–shRNA and *ABCC6–shRNA* cells. Densitometric analysis of the immunoreactive bands was performed in three independent experiments. The protein levels were normalized with β–actin content. Data were normalized to scr–shRNA cells set to 100%. Data are presented as the mean ± SEM of three independent experiments. (**C**) Representative images of immunofluorescence staining of N–cadherin, E-cadherin, and Vimentin (green signal) in fixed scr–shRNA HepG2 cells in comparison to *ABCC6–shRNA*. HepG2 cells using a 20× FLoidTM Cell Imaging Station fluorescence microscope. Cells were counterstained with 4′,6-diamidino-2-phenylindole (DAPI) to visualize nuclei (blue signal). Scale bar: 100 μm. (**D**) Expression level of extracellular Vimentin. The protein levels in the culture media of scrambled and silenced cells were normalized with total protein content. Data shown refer to those obtained on the culture medium of the scrambled cells set to 100%. Data are presented as the mean ± SEM of three independent experiments. Statistical analysis was performed using Student’s *t* test; * *p* < 0.05, ** *p* < 0.01.

Epithelial–Mesenchymal Transition (EMT) is a process in which tumor cells lose their epithelial characteristics and transform into more aggressive mesenchymal cells, and this process is a vital mechanism for tumor metastasis [34]. EMT frequently results in the loss of (or a reduction in) E-cadherin and an increase in Vimentin and N-cadherin expression levels, a crucial mechanism in causing cancer to migrate and invade. In contrast to the control cells, the *ABCC6* knockdown cells consistently showed up-regulation of the epithelial marker E-cadherin and down-regulation of the mesenchymal marker Vimentin. No changes were observed in the N-cadherin expression. Moreover, *ABCC6*-silenced cells showed a decreased level of extracellular Vimentin (Figure 9).

## 3. Discussion

*ABCC6* is highly expressed in HepG2 cells; therefore, it may have a role in controlling cell migration and invasion by feeding the extracellular purine pool with ATP and adenosine (the end product of ATP degradation) via ectonucleotidases such as CD39 and CD73 [5,25].

The role of ABCC6 in cancer has often been controversial; therefore, to further understand its role in hepatoma biology, we investigated the effect of *ABCC6* gene silencing on the HepG2 cells transcriptome. Differential expression analysis of mRNA transcripts between *ABCC6*-silenced HepG2 and scrambled cells revealed 476 statistically significant genes. Among them, 256 genes were significantly up-regulated and 208 genes were significantly down-regulated. GO enrichment analysis showed that the up- and down-regulated differentially expressed genes were mainly related to the mitochondrial matrix, cell surface, and DNA–binding transcription factor binding. The KEGG enrichment analysis showed that the differentially expressed genes participated in 91 statistically significant pathways, mainly dilated cardiomyopathy, focal adhesion, and proteoglycans.

Focal adhesions connect ECM to the cell cytoskeleton and participate in the early stages of the metastasis process, a sequential, multi-stages process which comprises the reorganization of cell–ECM interaction, matrix destruction and invasion, and new tumor formation in a secondary site [35,36,37].

RNA-seq analysis highlighted the up-regulation of the ITGA2 and ITGA6 integrins, which are responsible for cell–cell and cell–matrix adhesion as long as they can activate both “inside-out” signaling, thus promoting cell migration and ECM assembly and remodeling, as well as “outside-in” signaling, changing cytoskeletal structure [38,39,40]. In silenced *ABCC6* cells, we observed the overexpression of integrins ITGA2 and ITGA6 and, as a consequence, the adhesion of the silenced cells to Matrigel, an excellent substitute for the ECM for in vitro studies, significantly increased. Therefore, ABCC6 could be involved in the adhesion dynamics between cells and matrix cells, modulating the ability of HepG2 cells to adhere to the ECM.

The transwell migration and invasion assay shows that silencing *ABCC6* modifies the capacity of HepG2 cells to move and invade. The low ability of *ABCC6*-silenced cells to invade the Matrigel was most likely due to the reduced expression and secretion of active forms of the two metalloproteinases MMP2 and MMP9, whose activities have been correlated with the invasive stage of carcinomas [33,41].

Very aggressive tumor cells with a mesenchymal character have the unique capacity to migrate from the primary site and create a new tumor in other organs. The acquisition of this aggressive phenotype during epithelial–mesenchymal transition (EMT) involves the up-regulation of N-cadherin and Vimentin, followed by the down-regulation of E-cadherin [42,43,44,45,46]. *ABCC6* knockdown results in the over-expression of the epithelial marker E-cadherin and the down-regulation of the mesenchymal markers Vimentin and N-cadherin, thus confirming the lower propensity for aggressiveness of the *ABCC6*-silenced cells. Even the decreased ability to survive over time and expand into a clonal population when seeded at a low density supports *ABCC6*-silenced cell limited aggressive behavior, according to a senescent phenotype that has already been demonstrated and discussed [47].

The down-regulation of FLNC and MATN2 are also in agreement with this phenotype. FLNC and MATN2 are two proteins that help to remodel the cytoskeleton, influencing cell migration and invasion. FLNC is a cytoskeletal protein that has been identified as a potential hepatocellular carcinoma progression marker; altered FLNC expression may lead to enhanced tumor cell motility and invasiveness [48,49]. MATN2 is an extracellular matrix protein involved in the formation of filamentous networks; its expression is increased in hepatocellular carcinoma and liver cirrhosis [50,51].

Overall, the effects observed following *ABCC6* silencing in the HepG2 cells could be both the direct result of the modulation of intracellular metabolic pathways and the involvement of the purinergic system. In *ABCC6*-silenced HepG2 cells, we previously observed a decrease in the p-AKT/AKT and p-ERK/ERK ratios [25,26]. The activation of purinergic receptors in control cells by available extracellular ATP would trigger the intracellular signaling pathways of phosphatidylinositol 3-kinase/AKT (PI3K/AKT) and mitogen-activated protein kinase/extracellular signal-regulated kinase (MAPK/ERK), which, in turn, would recruit downstream effectors, which can ultimately affect adhesion, migration, and invasion by controlling the expression of related genes. The involvement of these signaling pathways and their implications for liver cancer are widely documented [52,53,54]. As a result, it is likely that the effects observed in the knockdown cells are precisely due to the reduction in extracellular ATP resulting from the deregulation of ABCC6.

There is no doubt that the enormous amount of data available from transcriptomic analysis opens up the possibility of investigating any other not yet explored role of ABCC6, which might assume the role of a regulator in the biology of hepatocellular carcinoma. In addition, the findings of this study could make an important contribution to the search for effective drugs for the management of hepatocellular carcinoma, for which there are still limited therapeutic options, as most are based on tyrosine kinase inhibitors, which mostly show poor efficacy and numerous collateral effects [55]. Few studies have proposed the use of prodrugs as a more tolerated and safer therapeutic alternative [56,57].

The concomitant administration of ABCC6 inhibitors and tyrosine kinase inhibitors has been suggested as a therapeutic option for the treatment of chronic myeloid leukemia [12], and it could certainly be interesting to evaluate its potential on hepatoma cell cultures. Furthermore, the intricate interplay between all the pathways regulated by ABCC6 and the dysregulated genes could allow us to hypothesize about using other molecules with anti-tumor activity in combination with ABCC6 inhibitors.

Although the data obtained from this study cannot be directly translated to what happens in vivo to tumor cells, as the tumor microenvironment is a much more complex system of the immune and stromal cells which interact with cancer cells, it would be interesting to investigate the role of ABCC6 in relation to the antitumor responses and immune evasion mechanisms in HCC. Our future studies will center around investigating this aspect.

## 4. Materials and Methods

### 4.1. Cell Lines and Maintenance of Cell Culture

The human hepatocellular carcinoma cell lines (HepG2) were purchased from ATCC (American Type Culture Collection) and maintained in Dulbecco’s modified Eagle’s medium (DMEM) containing 4.5 g/L glucose and supplemented with 10% fetal bovine serum (FBS), 2 mM L-glutamine, 60 µg/mL penicillin G, and 100 µg/mL streptomycin sulfate (EuroClone S.p.A, Pero, Italy). Cells were incubated in a humidified incubator at 37 °C under an atmosphere containing 5% CO_2_. The cells were subcultured at preconfluent densities using 0.25% trypsin-EDTA (EuroClone S.p.A, Pero, Italy).

### 4.2. Gene Knockdown of Stable Cell Lines

The *ABCC6* knockdown HepG2 cell line was constituted using a lentivirus shRNA knockdown vector system. Cell transfection was carried out according to the instructions of the manufacturer, VectorBuilder Inc. (Chicago, IL, USA), from whom the lentiviruses were purchased. Cells were transfected with viral particles containing a mixture of three different shRNAs, with each one targeting a different area of the *ABCC6* transcript: shRNA#1 AGATCGAGTTCGGGACTTTG targeted sequences (nucleotides 3773-3919) within exon 26; shRNA#2 CAACAAGGCAATAGCATTTAA targeted sequences within exon 6 (nucleotides 700–831) by exon 7 (nucleotides 832–1035); shRNA#3 TCCCTGCCTCCACAGAATAAA targeted sequences within exon14 (nucleotides 1905–1980) by exon 15 (nucleotides 1981–2107). Scramble–shRNA (scr–shRNA) CCTAAGGTTAAGTCGCCCTCG (used as control) does not map with *ABCC6*. HepG2 cells were seeded at a density of 1.5 × 10^4^ in a 12-well culture plate. After 24 h, the cells were transfected with a mixture of the three *ABCC6–shRNAs* or scr–shRNA as a control. The HepG2 cells were selected with 2 μg/mL puromycin for 12 days in order to select stable silenced cells. After selection, individual resistant clones were expanded in medium without puromycin, and the clones silenced between 70% and 80% were used for the experiments (Supplementary Figure S1). Three different clones of scrambled and *ABCC6*-silenced cells were used for biological replicates.

### 4.3. RNA Sequencing

The total RNA was extracted from the cells by using the Quick-RNA MiniPrep kit (ZymoResearch, Irvine, CA, USA) according to the manufacturer’s instructions. RNA was stored at −80 °C until further processing. RNA concentration and purity were evaluated using NanoDrop™ 2000/2000c (Thermo Fisher Scientific, Waltham, MA, USA), whereas sample integrity was analyzed using Tape Station 4200 (Agilent Technologies, Santa Clara, CA, USA) using an RNA Screen Tape Assay. Indexed libraries were prepared from 1 µg/ea purified RNA using the TruSeq Stranded mRNA (Illumina, San Diego, CA, USA) Library Prep Kit according to the manufacturer’s instructions.

After the enrichment of mRNA using oligo dT magnetic beads and fragmentation, cDNA synthesis was performed, followed by adapter ligation and PCR amplification. For library quantifications, TapeStation 4200 (Agilent Technologies) was used. Indexed libraries were pooled in equimolar amounts, with a final concentration of 2 nM.

The Illumina NextSeq 550 DX System was used to sequence the pooled samples in a 2 × 75 paired-end format. The raw sequence files generated (fastq files) underwent quality control analysis using FastQC [58]. Low-quality reads, short reads (≤25 bp), and adaptor sequences were trimmed using cutadapt (v.2.8) [59]. Then, the fastq files were mapped on the reference genome using the bioinformatics tool STAR (version 2.7.3a) [60] with the standard parameters for paired reads. The reference track was the Human assembly obtained from GenCode (HG38-Release 37 (GRCh38.p13) [32].

The quantification of genes expressed for each sequenced sample was computed using the featureCounts algorithm [61]. An ad hoc script in R was used to normalize the data using negative binomial generalized linear models, considering all genes expressed in the samples using the Bioconductor DESeq2 package [62]. Genes showing fold change ≥ 1.50 or ≤−1.50 (|FC| ≥ 1.50), along with adjusted *p* values ≤ 0.05 (padj), were considered as differentially expressed. The ComplexHeatmap [63,64] and ggplot2 [65] package in R were used to create heat maps and volcano plots of the differentially expressed genes, respectively.

### 4.4. Functional and Pathway Analysis of Differentially Regulated Genes

The R package pathfinder [66] intended for the identification of enriched pathways was used to perform the functional analysis. In particular, pathfindR analysis was based on the KEGG pathway database and GO database, and selected genes were set with padj ≤ 0.05 and |fold-change| ≥ 1.5. Only the enriched terms with adjusted *p* value ≤ 0.05 were used for the downstream analysis, including the hierarchical clustering of the terms. Furthermore, the pathfindR function score terms were used to calculate the aggregated term scores per sample based on gene expression patterns.

### 4.5. Real-Time Reverse Transcription Polymerase Chain Reaction (RT-qPCR)

The silenced and control cells were harvested, and total RNA was extracted using the Quick-RNA MiniPrep kit (Zymo Research, Irvine, CA, USA) according to the manufacturer’s protocol. cDNA was synthesized using a High-Capacity cDNA Reverse Transcription Kit (Applied Biosystem, Foster City, CA, USA) in accordance with the manufacturer’s instructions. Real-time quantitative RT-PCR was performed via a 7500 Fast Real-Time PCR System (Applied Biosystems) using iTaqTM Universal-SYBR^®®®®^ Green Supermix (Bio-Rad). To confirm PCR specificity, the PCR products were subjected to a melting curve analysis. RT-PCR results are expressed as the 2^−ΔCt^, with β-actin as the endogenous reference control. Primers were designed to span exon–exon junctions, eliminating undesirable genomic DNA amplification (Table 3).

### 4.6. Western Blot Analysis

The cells were suspended in radioimmunoprecipitation assay (RIPA) buffer (0.1% sodium dodecyl sulfate, 1% NP-40, and 0.5% sodium deoxycholate in PBS at pH 7.4) supplemented with a protease and phosphatase inhibitor cocktail (Roche, Penzberg, Germany) and lysed via sonication. Then, the lysates were centrifuged at 13,000 rpm for 10 min at 4 °C. The proteins (40 μg) were resuspended in Laemmli sample buffer (60 mM Tris–HCl pH 6.8, 10% glycerol, 2% SDS, 1% β-mercaptoethanol and 0.002% bromophenol blue), loaded into sodium dodecyl sulfate–polyacrylamide gels electrophoresis, and electrophoretically transferred to nitrocellulose membranes (Amersham Bioscience, Buckinghamshire, UK). The membranes were blocked in a saturation buffer (with 5% non-fat milk in PBS with 0.05% Tween 20, PBST) for 2 h at room temperature and then probed overnight at 4 °C with specific primary antibodies: 1:10,000 anti-β-actin monoclonal antibody (cat no. MA1-140; Thermo Fisher Scientific, Inc., Waltham, MA, USA); 1:5000 E-cadherin Monoclonal antibody (cat no. 60335-1-Ig; ProteinTech Group, Inc., Chicago, IL, USA); 1:1000 MMP9 (N-terminal) Polyclonal antibody (cat no. 10375-2-AP, ProteinTech Group, Inc.); 1:1000 MMP2 Mouse Monoclonal antibody (cat no. 66366-1-Ig; ProteinTech Group, Inc., Chicago, IL, USA); 1:5000 N-cadherin Monoclonal antibody (cat no. 66219-1-Ig, ProteinTech Group, Inc.); 1:1000 Cadherin-17 Polyclonal antibody (cat no. 24339-1-AP, ProteinTech Group, Inc.); 1:1000 Matrilin 2 Polyclonal antibody (cat no. 24064-1-AP, ProteinTech Group, Inc.); 1:1000 FLNC Polyclonal antibody (cat no. 28492-1-AP, ProteinTech Group, Inc.); 1:5000 Vimentin Mouse Monoclonal antibody (cat no. 10366-1-AP, ProteinTech Group, Inc.). The membrane was washed three times with PBST and then incubated at room temperature for 1 h with an appropriated horseradish peroxidase-conjugated secondary antibody and signal visualized via ECL™ Western Blotting Detection Reagents (GE Healthcare, Chicago, IL, USA) or the SuperSignal™ West Pico PLUS Chemiluminescent Substrate (Thermo Scientific, Waltham, MA, USA) using a Chemidoc™ XRS detection system equipped with Image Lab 5.1 software for image acquisition (BioRad). Densitometric analysis was performed by using GelAnalizer 19.1 (Istvan Lazar, www.gelanalyzer.com (accessed on 1 January 2023). The protein expression level in the control sample was taken as 100%. Each result was expressed as a percentage of the value of the control sample. Each test was repeated three time.

### 4.7. Cell Adhesion Assay

The *ABCC6–shRNA* cells and control cells (5 × 10^6^ cells/mL) were resuspended in serum-free medium containing 5 μM Calcein AM (acethoxymethyl ester). After incubation at 37 °C for 30 min, the cells were washed with PBS and resuspended in their respective medium, and 100 μL of cell suspension was added to plates coated for 120 min with 100 μg/mL of Matrigel, an extract derived from a tumor, containing all of the major components of many tissue basement membranes (Corning Life Sciences, Shanghai, China). Non-adherent cells were removed by gentle washing with PBS, and the fluorescence emission of adherent cells was monitored at 520 nm. The fluorescence signal of the cells before the two hours of incubation was designated as 100% control. The percentage of adhesion was calculated by dividing the corrected fluorescence (subtracted from the background) of the adhering cells by the corrected total fluorescence of the cells added to each corrected microplate (background subtracted) and multiplying by 100%

### 4.8. Migration and Invasion Assays

The *ABCC6–shRNA* and controls cells were maintained in serum-free medium for 24 h and then added to the upper chamber of a non-coated 24-well transwell plate (pore size: 8 µm, Sterlitech Corporation, Auburn, WA, USA) for the migration assay. In the cell invasion experiment, the transwells were coated with Matrigel 100 μg/mL (Corning Life Sciences, Shanghai, China). DMEM supplemented with 10% FBS was added to the lower chamber as a chemoattractant. Following incubation for 48 h at 37 °C, the cells were fixed with 4% paraformaldehyde for 10 min at room temperature and stained for 10 min with 0.1% crystal violet. Cotton swabs were used to remove non-invading cells from the upper surface. The cells were photographed under a light microscope. Experiments were conducted in triplicate, and the percentages of migrated and invasive cells are expressed herein as mean ± SEM.

### 4.9. SDS–Gelatin Gel Zymogram

Samples of freeze-dried serum-free medium (50 µg of proteins) of silenced and control cells were added to non-reducing sample buffer (50 mM Tris–HCl pH 6.8, 10% glycerol, 4% SDS, 0.002% bromophenol blue) and fractionated via electrophoresis using 8% SDS-polyacrylamide gels containing copolymerized gelatin 1 mg/mL. The gels were then washed twice with Triton X-100 2.5% and incubated overnight at 37 °C in buffer 50 mM Tris–HCl pH 6.8, 10 mM CaCl_2,_ and 1% Triton X-100. The gels were stained with 0.25% Coomassie^®®®®^ Brillant Blue R-250 (Sigma) and 0.05% Coomassie^®®®®^ Brillant Blue G-250 (Sigma), and MMP activities were detected as transparent bands on the blue background.

### 4.10. Colony Formation Assay

The *ABCC6–shRNA* cells and control cells were seeded in triplicate on a six-well plate with 500 cells/well and cultured at 37 °C and 5% CO_2_. After two weeks, colonies were then fixed with 4% paraformaldehyde and stained for 30 min with 0.1% crystal violet before being washed with phosphate-buffered saline (PBS). The colonies were counted under a microscope (Nikon Eclipse TS100).

### 4.11. Statistical Analyses

All of the assays were performed at least three times independently. Statistical analyses were performed using Student’s *t*-test. Where indicated, data are presented as the means ± SEM, as determined using GraphPad Prism 8 software (GraphPad Software, San Diego, CA, USA). * *p* < 0.05, ** *p* < 0.01, and *** *p* < 0.001 were considered to be statistically significant.

## Figures and Tables

**Table 1:** The top 10 differentially expressed genes (up-regulated and down-regulated) in *ABCC6*-silenced HepG2 cells.

Gene Symbol	Fold Change	padj	Regulation
MATN2	−6.52	2.08 × 10^−4^	
ABCG8	−6.48	1.55 × 10^−2^	Down
PITPNM3	−6.46	9.42 × 10^−3^	
FADS6	−5.71	1.60 × 10^−2^	
FLNC	−5.64	5.94 × 10^−7^	
JAM3	−5.12	3.35 × 10^−2^	
ICAM2	−4.88	8.39 × 10^−3^	
UNC13C	−4.66	5.54 × 10^−4^	
PLCB2	−4.63	5.55 × 10^−4^	
SLC22A7	−4.46	4.40 × 10^−2^	
GLIPR1	6.14	2.30 × 10^−5^	
NXPH3	6.63	1.62 × 10^−5^	Up
ISM1	6.99	1.50 × 10^−10^	
CDH17	7.68	1.94 × 10^−3^	
RAB27B	7.72	1.32 × 10^−3^	
RIPPLY3	8.08	4.54 × 10^−10^	
PAUPAR	11.81	3.53 × 10^−4^	
LYVE1	16.55	4.57 × 10^−9^	
MMP7	19.08	1.17 × 10^−17^	
DHRS9	22.97	2.90 × 10^−5^	

Fold Change = ratio of the normalized expression absolute value in sample over control; padj = *p* value adjustment.

**Table 2:** The top 10 enriched signaling pathways of up-regulated and down-regulated differentially expressed genes.

ID	Term Description	Fold- Enrichment	Occurrence	Support	Lowest_p	Highest_p	Up_Regulated	Down_Regulated
hsa05414	Dilated cardiomyopathy	5.36	10	0.12	5.41 × 10^−9^	3.54 × 10^−7^	ITGA2, ITGA6, ITGB1, ACTG1, TPM4, ADCY7, TGFB1	ITGA3, TTN, ADCY9
hsa04510	Focal adhesion	3.36	10	0.14	1.68 × 10^−6^	8.16 × 10^−6^	LAMB3, SPP1, ITGA2, ITGA6, ITGB1, ARHGAP5, ACTG1, CAPN2, FLNA	VTN, ITGA3, PPP1CC, FLNC
hsa05410	Hypertrophic cardiomyopathy	4.58	10	0.09	3.04 × 10^−7^	9.07 × 10^−6^	ITGA2, ITGA6, ITGB1, ACTG1, TPM4, TGFB1	ITGA3, TTN
hsa05205	Proteoglycans in cancer	3.26	10	0.14	3.28 × 10^−6^	1.23 × 10^−5^	ACTG1, FLNA, ITPR3, CD63, TGFB1, PLAUR, ITGA2, ITGB1, RDX	FLNC, PPP1CC, TIMP3, VTN
hsa05132	Salmonella infection	2.06	10	0.03	3.86 × 10^−5^	3.86 × 10^−5^	DYNLL2, DYNLRB1, TUBA1A, TUBB2A, FLNA, ACTG1, SNX9	FLNC, CYFIP2, CD14
hsa04610	Complement and coagulation cascades	5.99	10	0.04	4.46 × 10^−5^	4.46 × 10^−5^	F2R, SERPINE1, PLAUR	FGG, KNG1, MBL2, MASP2, C3, CLU, VTN
hsa05412	Arrhythmogenic right ventricular cardiomyopathy	4.02	10	0.07	2.07 × 10^−5^	4.53 × 10^−5^	ITGA2, ITGA6, ITGB1, ACTG1, DSP	ITGA3
hsa04512	ECM–receptor interaction	4.63	10	0.05	2.09 × 10^−5^	8.19 × 10^−5^	LAMB3, SPP1, NPNT, ITGA2, ITGA6, ITGB1	VTN, ITGA3
hsa05145	Toxoplasmosis	2.27	10	0.03	8.49 × 10^−5^	1.02 × 10^−4^	LAMB3, ITGA6, ITGB1, LDLR, TGFB1	
hsa05222	Small-cell lung cancer	3.32	10	0.05	2.61 × 10^−5^	1.02 × 10^−4^	CDKN2B, LAMB3, ITGA2, ITGA6, ITGB1	ITGA3

ID: ID of the enriched term; Term_Description: description of the enriched term; Fold_Enrichment: fold enrichment value for the enriched term; Occurrence: the number of iterations that the given term was found to be enriched in over all iterations; Lowest_p: the lowest adjusted *p* value of the given term over all iterations; Highest_p: the highest adjusted *p* value of the given term over all iterations; Up_regulated: the up-regulated genes (as determined by change value > 0, if the change column was provided) in the input involved in the given term’s gene set, comma-separated. If change column was not provided, all affected input genes are listed here. Down_regulated: the down-regulated genes (as determined by change value < 0, if the change column was provided) in the input involved in the given term’s gene set, comma-separated.

**Table 3:** List of primers used in this study.

Gene	Accession Number	Forward Primer	Reverse Primer
β-actin	NM_001101.3	5′-CCTGGCACCCAGCACAAT-3′	5′-GCCGATCCACACGGAGTACT-3′
*ABCC6*	NM_001171.5	5′-AAGGAACCACCATCAGGAGGAG-3′	5′-ACCAGCGACACAGAGAAGAGG-3′
CDH1	NM_001317184.2	5′-CTCCCTTCACAGCAGAACTAACAC-3′	5′-GTCCTCTTCTCCGCCTCCTTC-3′
CDH2	NM_001308176.2	5′-GGATCAAAGCCTGGAACATAT-3′	5′-TTGGAGCCTGAGACACGATT-3′
CDH17	NM_004063.4	5′-TCAAAATCACTCAGGTGCGG-3′	5′-GAAAAATGGGAATCTTGGGAGC-3′
FLNC	NM_001458.5	5′-CTGTCCATGTGTCGGAAGCC-3′	5′-ACACCTTGAAGTCAGCCACC -3′
ITGA2	NM_002203.4	5′-AAATGATATTCTGATGCTGGG-3′	5′- CCA GCC TTT TCT AGT AGA GC-3′
ITGA6	NM_000210.4	5′-CTT AGG TTT TTC TTT GGA CTC A-3′	5′-TCTCTTCAGCAAAACCACGG-3′
MATN2	NM_002380.5	5′-AAACGCTGCCGAAGGAAGG -3′	5′-TCCTCAGCTAGAACAAATCCG -3′
VIM	NM_003380.5	5′-ATGGACAGGTTATCAACGAAA-3′	5′-AAGTTTGGAAGAGGCAGAGA-3′

β-actin, Beta Actin; ABCC6, ATP-binding cassette subfamily C member 6; CDH1, Cadherin 1 or E-cadherin; CDH2, Cadherin 2 or N-cadherin; CDH17, Cadherin 17; FLNC, Filamin C; ITGA2, Integrin Subunit Alpha 2; ITGA6, Integrin Subunit Alpha 6; MATN2, Matrilin 2, VIM, Vimentin.

## Data Availability

The data presented in this study are available from the corresponding author upon request.

## Supplementary Materials

The following supporting information can be downloaded at: https://www.mdpi.com/article/10.3390/ijms242216391/s1.
