# Supplementary material for: A Regulator Role for the ATP-Binding Cassette Subfamily C Member 6 Transporter in HepG2 Cells: Effect on the Dynamics of Cell–Cell and Cell–Matrix Interactions
Source: Int J Mol Sci. 2023 Nov 16;24(22):16391. doi: 10.3390/ijms242216391 (PMC10670978; doi:10.3390/ijms242216391)
Supplement: Supplementary file 1 [file ijms-24-16391-s001.zip › ijms-2703200-Figure S1.docx]

**Supplementary Figure S1**

| **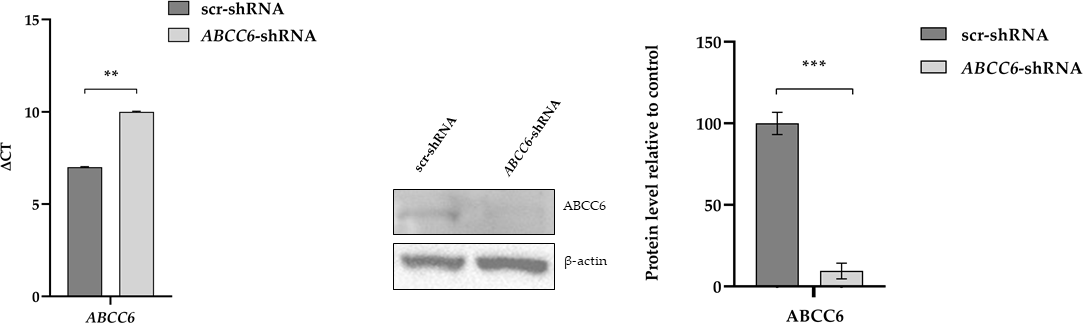** | |
| --- | --- |
| (**A**) | (**B**) |

**Supplementary Figure S1.** (**A**) *ABCC6* gene expression in *ABCC6*-shRNA and scr–shRNA HepG2 cells. Expression was normalized to β–actin mRNA levels (ΔCt). ** *p* < 0.01 ABCC6–shRNA HepG2 cells vs. scrambled HepG2 cells; (**B**) Representative western blot and densitometric analysis of the immunoreactive bands. The protein levels were normalized with β–actin content and referred to that of scr–shRNA cells set to 100%. Data are presented as mean ± SEM of at least three independent experiments. Statistical analysis was performed by Student’s t test; *** *p* < 0.001.
